# Supplementary material for: Resistance Training Reshapes the Gut Microbiome in a Longitudinal 8-Week Intervention in Sedentary Adults
Source: Sports Med Open. 2026 Mar 16;12:21. doi: 10.1186/s40798-026-00990-6 (PMC12989468; doi:10.1186/s40798-026-00990-6)
Supplement: Supplementary file 1 — Supplementary Material 1. [file 40798_2026_990_MOESM1_ESM.pdf]

Title: Resistance Training Reshapes the Gut Microbiome in a Longitudinal 8-Week Intervention in Sedentary Adults

Authors: Daniel Straub[1,2], Till Englert[1,2], Antonia Beller[1], Josua Stadelmaier[1,2], Mark Stahl[3], Joachim Kilian[3], Jens Borzym[4], Carola Rotermund[5], Tanja Akbuga-Schön[5], Sabrina Krakau[1,2], Stefan Czemmel[1,2], Sabine Weiler[5], Marc Pettenkofer[4], Jörg Pettenkofer[4], Ulli Maser[4,], Sascha Dammeier[5], Andreas M. Nieß[6,7], Markus D Enderle[5] and Sven Nahnsen[1,2,8,*]

Affiliations: [1]Quantitative Biology Center (QBiC), University of Tübingen, Germany, [2]M3 Research Center, Medical Faculty, University of Tübingen, Germany, [3]Zentrum für Molekularbiologie der Pflanzen (ZMBP), University of Tübingen, Germany, [4]Fitness-Park Mapet GmbH, Tübingen, Germany, [5]Erbe Elektromedizin GmbH, Tübingen, Germany, [6]Department of Sports Medicine, University Hospital Tübingen, 72076 Tübingen, Germany, [7]Interfaculty Research Institute for Sport and Physical Activity, Eberhard Karls University Tübingen, Tübingen, Germany, [8]FBI/IBMI - Biomedical Data Science, University of Tübingen, Germany

***Corresponding author information:** Sven Nahnsen sven.nahnsen@qbic.uni-tuebingen.de

1. SupplementaryTables

Suppl. Table 1: Strength, fitness, demographic, and dietary information for all study participants or stratified by training or average strength gain subset. Values are presented as percent or mean ± SD, n is the number of valid values. Counts and means were compared between training types or low and high strength gain subgroups. Means were compared between training types with Kruskal-Wallis rank sum test and between responder subsets using Dunn's test of multiple comparisons using rank sums (R v4.2.3, R package "dunn.test" v1.3.6), respectively. Counts were compared with Pearson's Chi-squared test in R v4.2.3. P-values were corrected with Benjamini-Hochberg method to false discovery rates (fdr). #: 0 – never, 1 – less than once per week, 2 – 1-2x per week, 3 – 3-4x per week, 4 – 4-6x per week, 5- daily, 6 – multiple times a day. ##: calculated. Bold: fdr ≤ 0.05.

Measure	All participants		Training					Average strength gain				
			Regular resistance training		Muscle-building resistance training		fdr	low		high		fdr
	Value	n	Value	n	Value	n		Value	n	Value	n	
**BioAge – core [years]**	49.7±15.6	150	48.8±15.2	73	50.5±16.1	77	0.85	41.6±14.8	30	63±11.2	30	**0.000004**
**BioAge – strength [years]**	46.6±15.4	150	45.8±14.7	73	47.4±16	77	0.85	40.1±12.8	30	60.7±12.1	30	**0.000008**
**BioAge – legs [years]**	47.3±18.5	150	46.5±17.7	73	48.1±19.2	77	0.95	39.4±15.5	30	63±14.8	30	**0.000025**
**Strength – leg curls [kg]**	38.5±12.2	150	39.1±11.5	73	37.9±12.9	77	0.85	44.8±10.9	30	31.1±12.3	30	**0.000111**
**BioAge – upper body [years]**	42.7±16	150	41.9±15.6	73	43.5±16.5	77	0.89	39.2±13.9	30	55.9±14.1	30	**0.000551**
**Strength – abdominal trainer [kg]**	52.7±17.7	150	54±18.5	73	51.3±17	77	0.85	60.5±17.5	30	45.2±16.4	30	**0.000869**
**Strength – back trainer [kg]**	35.2±12.6	149	35.5±12.1	73	35±13.1	76	0.97	40.1±11	30	29.2±11.7	30	**0.000869**
**Strength – leg press [kg]**	154.5±53.9	150	157.4±50.9	73	151.7±56.8	77	0.85	174.5±44.7	30	128.2±59.7	30	**0.000869**
**Strength – lateral pulldown [kg]**	59.9±19.6	149	61.2±19.2	72	58.7±20.1	77	0.85	65.3±18.2	30	52±21.6	30	**0.010862**
**BioAge – total [years]**	49.1±11.6	148	48.8±11.3	71	49.3±11.9	77	0.97	46.7±11.7	30	55.2±10.1	30	**0.017507**
**Strength – rowing [kg]**	56.1±18.4	150	57.3±17.2	73	55.1±19.6	77	0.85	59.9±16.2	30	48.7±20.2	30	**0.019820**
**Food – fish#**	1.2±0.8	150	1.3±0.7	73	1.1±0.9	77	0.57	1±0.6	30	1.5±1	30	**0.049633**
Food – coffee [cups per day]	0.4±0.3	136	0.4±0.3	67	0.4±0.3	69	0.85	0.3±0.3	27	0.5±0.3	29	0.111597
Strength – chest press [kg]	50.4±18.8	150	50.3±19.1	73	50.5±18.6	77	1.00	52±15.2	30	45.3±17.8	30	0.173466
Food – water & tea [l per day]	1.7±0.9	147	1.7±0.9	70	1.7±0.9	77	1.00	2±1.1	29	1.5±0.6	30	0.173466
Health – BMI	24.5±3.5	150	24.7±3.7	73	24.4±3.4	77	0.97	24±3.2	30	25.3±3.2	30	0.196888
Food – eggs#	1.9±1	149	1.9±0.9	73	2±1	76	0.85	1.6±0.9	30	1.9±0.8	30	0.221492
Allergies – hay fever [%]	18.9%	148	15.5%	71	22.1%	77	0.85	10%	30	10%	30	0.223249
Health – body fat [%]	29.7±8	150	29.7±8.1	73	29.7±7.9	77	1.00	28.5±7.8	30	30.7±8	30	0.406275
Food – Juice & Lemonade [l per day]	0.3±0.4	127	0.3±0.4	62	0.2±0.3	65	0.64	0.3±0.4	28	0.2±0.3	28	0.406275
Height [cm]	173.5±9.5	147	173.8±9.4	71	173.3±9.6	76	0.97	176.3±10.3	30	173.5±8.3	30	0.406275
Activity – biking [km per week]##	13.1±22.5	145	14±24.9	71	12.2±20	74	0.97	11.1±18.5	28	12.3±15.3	30	0.406275
BioAge – metabolic [years]	58.2±17.3	148	58±16.5	71	58.4±18	77	1.00	57.3±18.5	30	61.1±15	30	0.406275

Allergies – lactose intolerance [%]	6.1%	148	9.9%	71	2.6%	77	0.64	13.3%	30	3.3%	30	0.406275
Medication – antibiotics treatment in last 12 months [%]	7.4%	148	2.8%	71	11.7%	77	0.57	0%	30	6.7%	30	0.406275
Medication – immunosuppressants ever received [%]	1.4%	144	0%	71	2.7%	73	0.85	3.4%	29	3.4%	29	0.470290
Health – heart rate [beats/min]	74.1±13.5	148	72.3±11.1	73	75.9±15.3	75	0.85	74.4±14.9	29	76.6±14.8	30	0.515132
Food – salty snacks[#]	1.8±1.1	150	1.8±1.1	73	1.7±1.1	77	0.85	1.7±1	30	1.8±1	30	0.515132
Age [years]	41.7±11.6	148	43.1±11.4	71	40.5±11.7	77	0.64	40.4±12.3	30	42.2±11.3	30	0.528696
Food – regular supplements [%]	18.9%	148	22.5%	71	15.6%	77	0.85	16.7%	30	10%	30	0.528696
Cigarettes in past [n per day]	0.5±3	150	0.4±3	73	0.6±3	77	1.00	1.2±4.5	30	0.5±2	30	0.529324
Stool samples at home [days][##]	0.6±0.8	148	0.8±0.9	71	0.5±0.8	77	0.57	0.7±0.9	29	0.8±1	30	0.530164
Food – dairy[#]	3.8±1.5	150	3.8±1.5	73	3.8±1.5	77	1.00	3.7±1.5	30	3.5±1.6	30	0.530164
Food – grains[#]	4.5±1.2	150	4.4±1.2	73	4.5±1.1	77	0.87	4.4±1.2	30	4.3±1.2	30	0.530164
Health – Calculated VO2 max [ml/kg/min]	38.3±7.1	149	38.1±7.6	72	38.5±6.7	77	0.85	38.1±5.5	29	37.7±8.2	30	0.536270
Food – meat[#]	2.2±1.2	150	2.3±1.2	73	2.1±1.3	77	0.85	2±1.1	30	2±1.5	30	0.536270
Health – Diastolic blood pressure [mmHg]	81±12.4	148	82.8±12.5	73	79.1±12.1	75	0.57	81.5±12	29	83.1±13.5	30	0.536270
Gender (male) [%]	42.6%	148	43.7%	71	41.6%	77	1.00	53.3%	30	43.3%	30	0.536270
BioAge – cardio [years]	42.4±9.4	148	42.7±10	71	42±8.8	77	0.97	43±8.7	30	43.8±10	30	0.542925
Food – sweets[#]	3.2±1.4	150	3.5±1.4	73	2.9±1.3	77	0.43	3±1.3	30	3.1±1.3	30	0.560570
Food – vegetable[#]	4.4±1.3	150	4.2±1.3	73	4.6±1.3	77	0.57	4.3±1.3	30	4.3±1.3	30	0.574921
Health – cigarettes currently [n per day]	0.4±1.6	150	0.2±0.9	73	0.6±2.1	77	0.64	0.7±2.8	30	0.3±1.2	30	0.574921
Food – alcohol [ml per week][##]	66.9±87.3	150	73.3±93.1	73	60.8±81.5	77	0.85	71.7±79.1	30	64.9±61.1	30	0.574921
Health – systolic blood pressure [mmHg]	125.7±18.3	148	127.7±17	73	123.7±19.4	75	0.57	125.6±17.2	29	127.9±21	30	0.574921
Weight [kg]	73.3±13	142	73.6±13.1	69	73±12.9	73	1.00	74.5±13.2	28	74.4±10.9	28	0.574921
Activity – Walking [km per week][##]	5.3±7.9	137	3.9±7.6	66	6.6±8	71	0.36	5.5±8.7	28	4.6±7.4	28	0.574921
Born in Germany [%]	82.4%	148	87.3%	71	77.9%	77	0.78	90%	30	80%	30	0.574921
Health - abnormal weight loss [%]	3.3%	150	2.7%	73	3.9%	77	1.00	6.7%	30	3.3%	30	0.574921
Food – any special diet [%]	17.3%	139	10.8%	65	23%	74	0.57	16.7%	30	24.1%	29	0.599666
Allergies – dust mites [%]	7.4%	148	7%	71	7.8%	77	1.00	3.3%	30	6.7%	30	0.631463
Health – active smoker [%]	23.3%	150	21.9%	73	24.7%	77	1.00	30%	30	23.3%	30	0.656136
Medication – laxative in last 6 month [%]	2%	147	1.4%	70	2.6%	77	1.00	0%	30	3.3%	30	0.672842
Health – abnormal weight change [%]	10.8%	148	9.9%	71	11.7%	77	1.00	13.3%	30	13.3%	30	0.743843
Allergies – any [%]	32%	150	27.4%	73	36.4%	77	0.85	33.3%	30	26.7%	30	0.796747
Medication – any [%]	24.7%	150	21.9%	73	27.3%	77	0.89	20%	30	26.7%	30	0.796747

Suppl. Table 2: Dietary and activity information for all study participants or average strength gain subset over time. Mean ± SD for start (week 0) and end (week 8) of the study, n is the number of complete cases (no missing value over time). Friedman test on all data (week 0, week 4, week 8) with p-values corrected to false discovery rates (fdr) using Benjamini-Hochberg method, no post-hoc or pairwise test was applied because all fdr were above the significance threshold of fdr=0.05. #: 0 – never, 1 – less than once per week, 2 – 1-2x per week, 3 – 3-4x per week, 4 – 4-6x per week, 5- daily, 6 – multiple times a day. ##: calculated, n.d.: not determined because of identical data (test produced no p-value).

	All participants						Average strength gain high responder			
	Total	n	Week 0	Week 8	n	fdr	Week 0	Week 8	n	fdr
Food – alcohol [ml per week]##	59.2±72.6	447	66.7±87.5	58.5±67.3	149	0.159	64±61.9	61±51.5	29	0.832
Health – Cigarettes in past [n per day]	0.3±2.2	447	0.5±3	0.2±1.5	149	0.159	0.5±2	0.4±1.9	29	0.832
Food – eggs#	2±1	444	1.9±1	2.1±1	148	0.159	1.9±0.9	1.9±1	29	0.963
Food – salty snacks#	1.7±1	447	1.8±1.1	1.6±0.9	149	0.171	1.8±1	1.5±1	29	0.832
Food – vegetable#	4.4±1.2	447	4.4±1.3	4.4±1.2	149	0.171	4.3±1.4	4.7±1.1	29	0.781
Food – Juice & Lemonade [l per day]	0.3±0.3	336	0.3±0.4	0.2±0.3	112	0.205	0.3±0.4	0.3±0.3	22	0.832
Food – coffee [cups per day]	0.4±0.2	372	0.4±0.3	0.4±0.2	124	0.411	0.5±0.2	0.4±0.2	25	0.781
Food – dairy#	3.8±1.5	447	3.8±1.5	3.7±1.5	149	0.411	3.4±1.6	3.5±1.7	29	0.892
Food – sweets#	3.1±1.3	447	3.2±1.4	3.1±1.4	149	0.742	3.1±1.3	3.1±1	29	0.963
Food – grains#	4.5±1.2	444	4.5±1.2	4.5±1.2	148	0.742	4.3±1.2	4.4±1	29	0.781
Food – fish#	1.2±0.8	447	1.2±0.8	1.1±0.8	149	0.796	1.6±1	1.5±1.1	29	0.963
Food – meat#	2.2±1.2	441	2.2±1.2	2.2±1.3	147	0.798	2±1.5	1.9±1.4	29	0.832
Health – cigarettes currently [n per day]	0.3±1.4	447	0.3±1.6	0.2±1.1	149	0.798	0.2±0.8	0±0.1	29	0.781
Food – water & tea [l per day]	1.7±1	435	1.7±0.9	1.7±0.9	145	0.798	1.5±0.7	1.5±0.7	28	0.832
Activity – Walking [km per week]##	5.3±7.9	411	5.3±7.9	5.3±7.9	137	n.d.	4.6±7.4	4.6±7.4	28	n.d.
Activity – biking [km per week]##	13.1±22.4	435	13.1±22.5	13.1±22.5	145	n.d.	12.3±15.3	12.3±15.3	30	n.d.

Suppl. Table 3: Prevalence of training types in responder type groups. Percentage of study participants that recieved regular resistance training (in contrast to muscle-building resistance training) for low, middle, high responder types or all 150 participants per subset group (leg press, average strength gain, BioAge). Pearson's Chi-squared test with p-values were corrected with Benjamini-Hochberg method to false discovery rates (fdr) (R v4.2.3).

Subset type	Responder type				Pearson's Chi-squared test			
	low	middle	high	all	df	X-squared	p-value	fdr
Leg press	43.3%	50.0%	50.0%	48.7%	2	1.2809	0.5271	0.5271
Avgerage strength gain	43.3%	52.2%	43.3%	48.7%	2	3.4158	0.1812	0.5271
BioAge	43.8%	51.1%	46.7%	48.7%	2	1.7176	0.4237	0.5271

2. Supplementary Figures

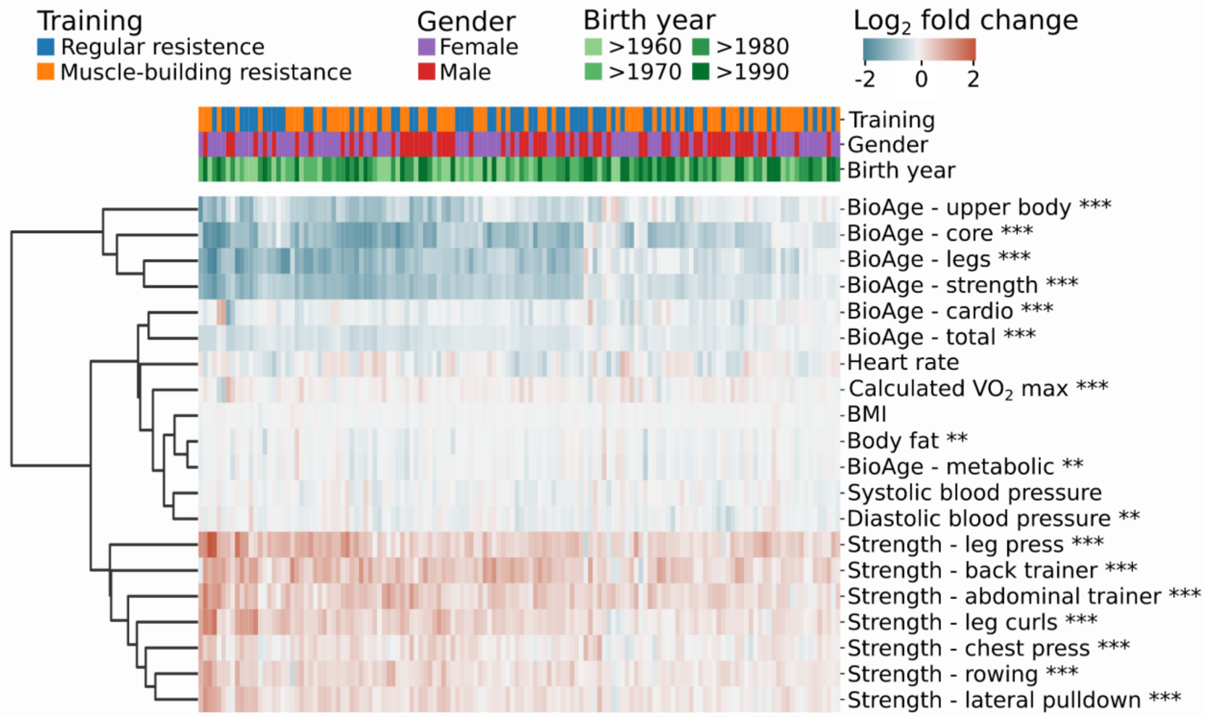

Suppl. Fig. 1: Heatmap of log_2 fold changes from weeks 0 to 8 of fitness and health related metrics clustered hierarchically by euclidean distance with significance indicated by asterisks. Non-parametric Friedman test for repeated measures (formula: compound ~ weeks + (1|participant)), Conover post hoc test, Benjamini-Hochberg adjusted p-values; *: p<0.05, **: p<0.01, ***: p<0.001

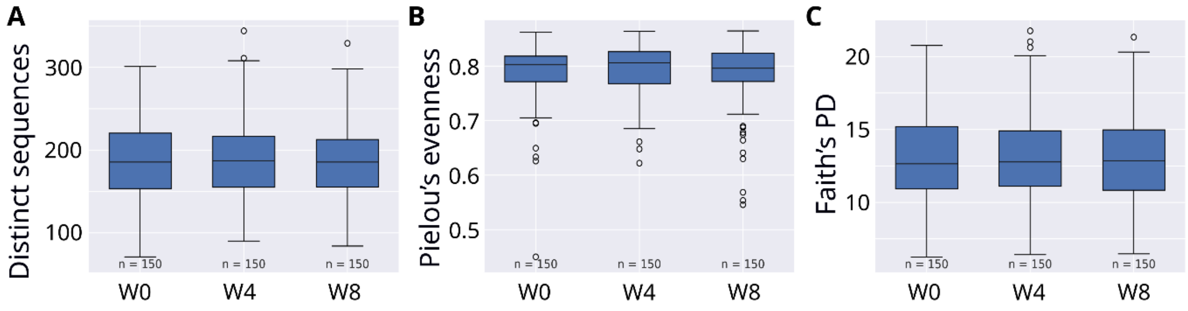

Suppl. Fig. 2: Alpha diversity indices at different time points. Number of distinct amplicon sequencing variants (ASVs) (A), Pielou's evenness (B), and Faith's phylogenetic diversity (Faith's PD) (C) over all study participants. Friedman test showed no significant differences between time points: distinct ASVs p=0.60, Pielou's evenness p=0.61, Faith's PD p=0.50.

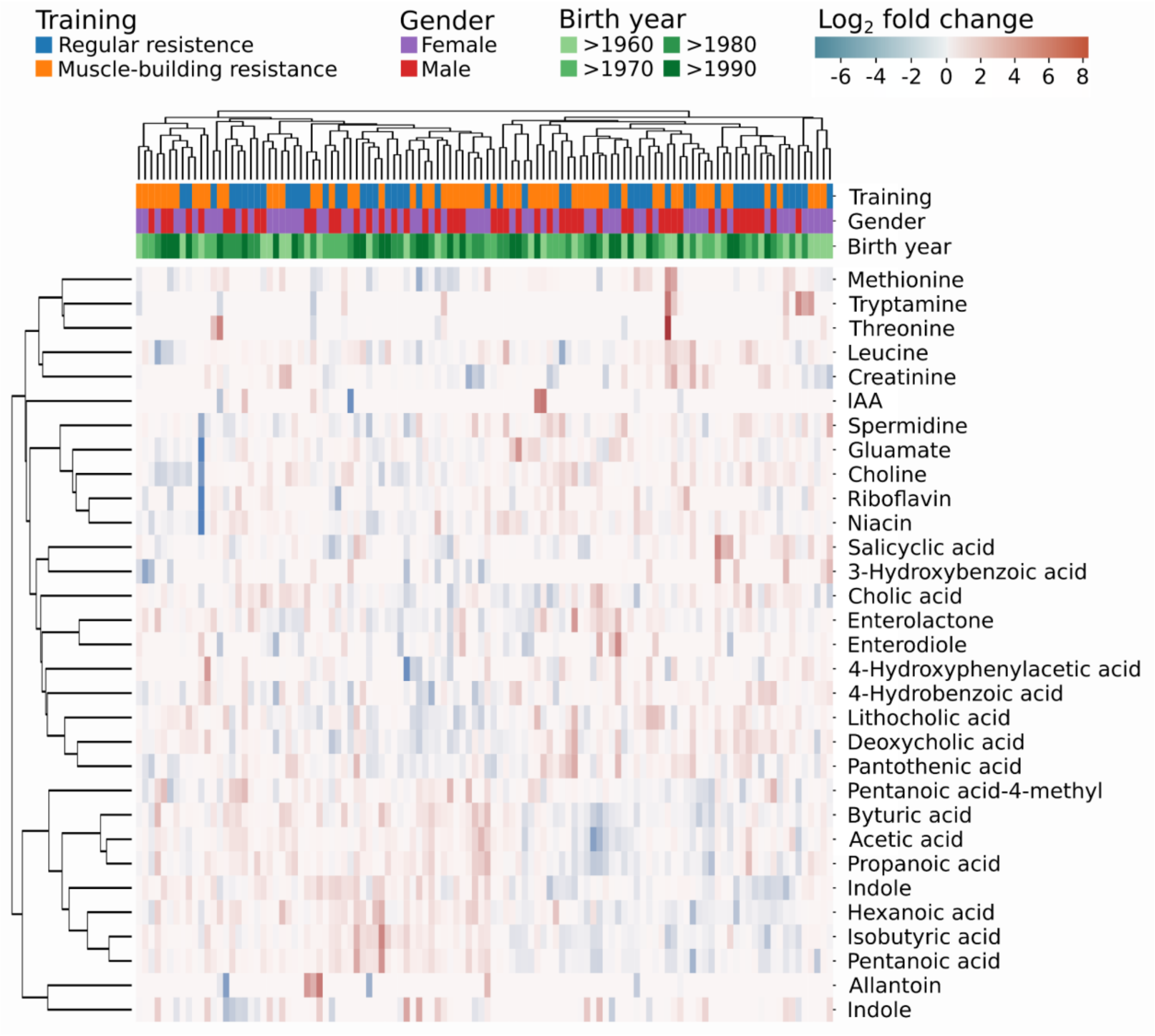

Suppl. Fig. 3: Heatmap of $\log_2$ fold changes from weeks 0 to 8 of metabolites clustered hierarchically by euclidean distance. No significant changes were detected using Friedman test adjusted with Benjamini-Hochberg to FDR or LME with model “metabolite ~ weeks + (1|Patient_id)”.

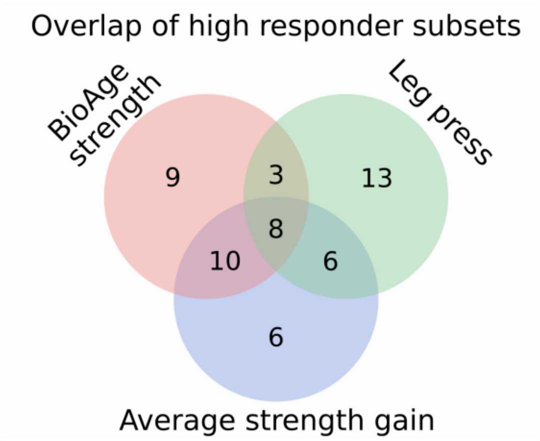

Suppl. Fig. 4: High responder (HR) subsets overlap partially. Participants were stratified into low-responder (LR, bottom 20%) and high-responder (HR, top 20%) across three strength metrics (Fig. 2C and Suppl. Fig. 4).

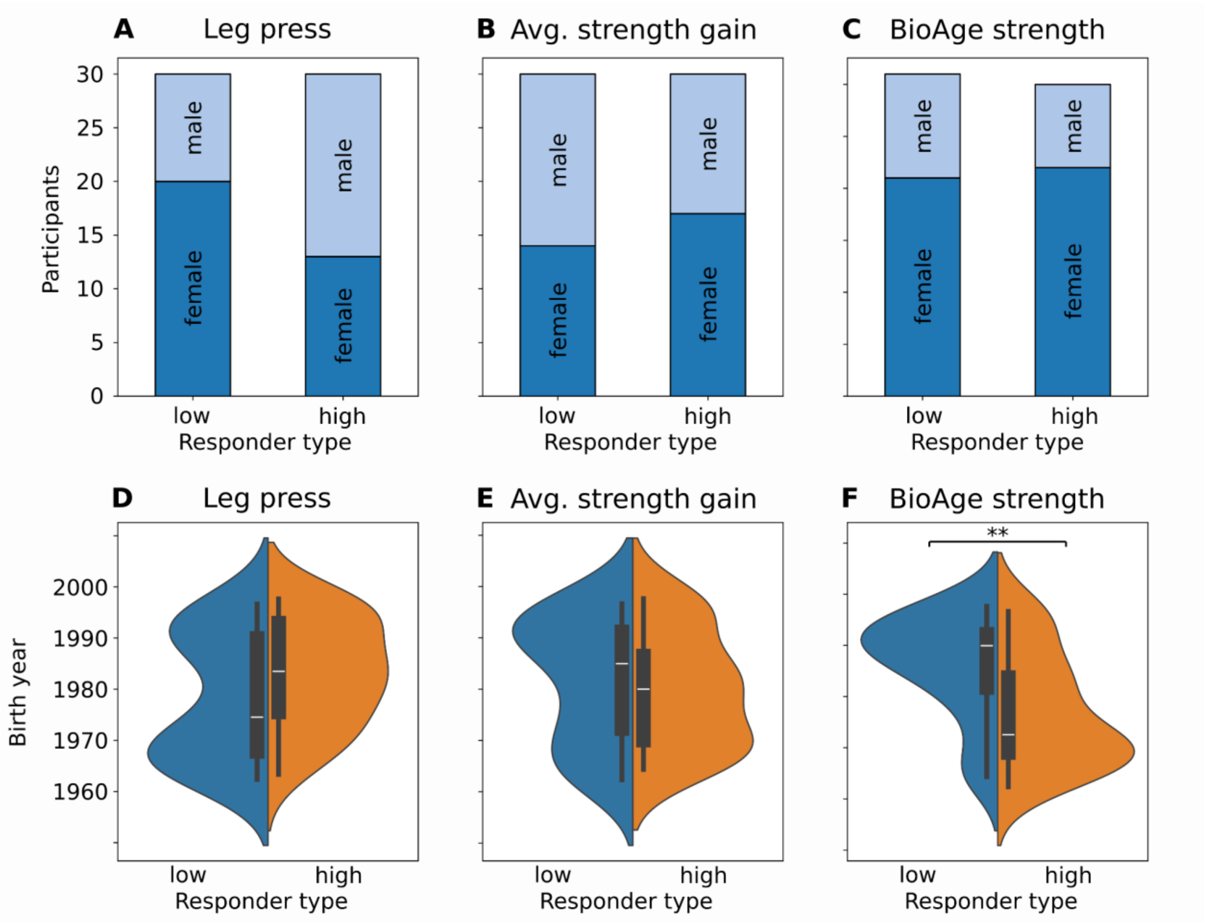

Suppl. Fig. 5: Demographics of low and high responders. Distribution of gender (A, B, C) and birth year (D, E, F) between low and high responder of maximum strength of leg press (A, D), average strength gain (B, E), or BioAge Strength (C, F). **: p≤0.01 using Kolmogorov-Smirnov test adjusted with Benjamini-Hochberg to FDR.

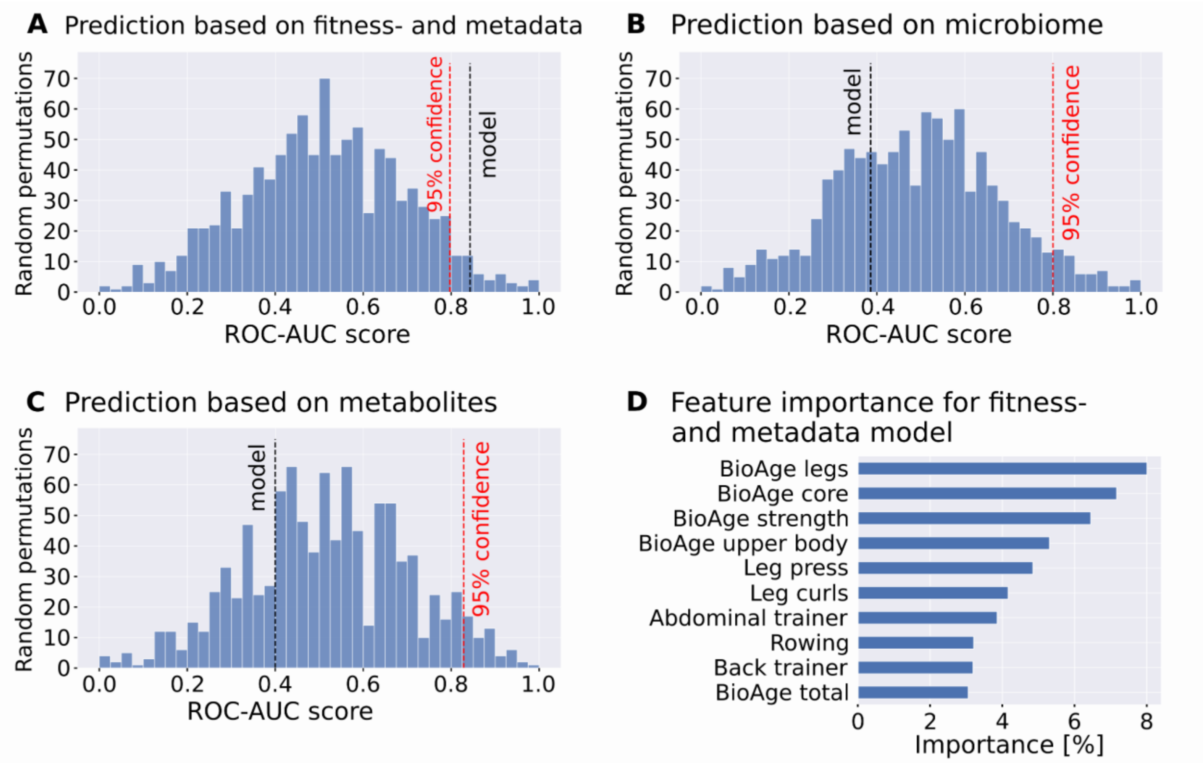

Suppl. Fig. 6: Prediction of low and high responder on all machines based on data collected at training start (baseline) with Random Forest. Model based on (A) fitness- and metadata - ROC-AUC=0.84, (B) microbiome - ROC-AUC=0.39, or (C) metabolites - ROC-AUC=0.40. (D) Ten most important features ("gini importance" [1]) in descending order of the prediction based on fitness- and metadata (i.e. model in A). Distribution of randomised training group predictions was achieved by permutation of the responder type (n=1000). Python v3.12.3 with scikit-learn v1.5.2.

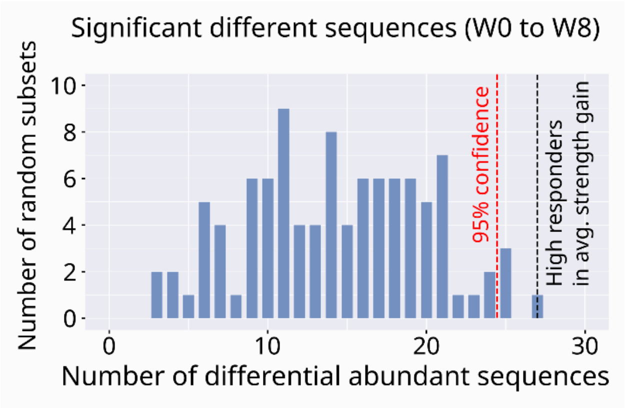

Suppl. Fig. 7: Distribution of number of significant different abundant 16S rRNA gene Amplicon Sequence Variants (ASVs) (log2fold change >=1 or <=-1 and p-value<=0.05) of 100 random subsets of non-high-responders compared to high responders in average exercises in support of Figure 4. High responders of average strength gain have more significantly different ASVs than 99 of 100 random subsets after 8 weeks (p=0.01). W0: week 0, W8: week 8.

3. Supplementary Methods

3.1. Strength and Training Metrics

The EGYM BioAge metric (referred to here as BioAge Total) is the average of three distinct BioAge components, including BioAge strength. In each of these, a lower BioAge indicates a healthier condition of the participant. Each BioAge component was calculated based on participant-specific data while accounting for sex differences. BioAge Metabolic is calculated based on body fat percentage, using a model informed by internal data and supported by previous literature describing the relationship between body fat and biological aging. BioAge Cardio is derived from estimated VO_2max values, with sex-specific calculations. VO_2max was estimated from maximal performance on cross trainers, using a model that combines internal performance data with previously validated methods [2–4]. The training instruments used are listed in Suppl. Table 4.

Suppl. Table 4: Instruments used

Device	Measurements/Calculations	Company	Product
Blood pressure monitor	Resting diastolic & systolic blood pressure, pulse rate	A & D Medical	UA-767PC
Scale	Body weight, percent body fat, BMI	Seca	Seca Tru Fitness (seca 552) - 10000000671720
Cross Trainer	VO_2 max	LifeFitness	Elevation Cross-Trainer - ASX127637
SMart Strength machines	Maximum strength	EGYM	Smart Strength Generation 2 (2015)

3.2. 16S rRNA gene amplicon sequencing

NGS sequencing methods were performed at the Institute for Medical Microbiology and Hygiene (MGM) of the University of Tübingen. About 200mg of each stool sample were resuspended in 800 µL CD1 buffer (DNeasy 96 PowerSoil Pro QIAcube HT Kit, QIAGEN) and transferred to a ZR BashingBead Lysis Tube (Zymo Research). The tube was vortexed horizontally for 10 minutes on a vortex shaker. DNA was then purified using DNeasy 96 PowerSoil Pro QIAcube HT Kit (QIAGEN) according to the manufacturer's instructions.

Genomic DNA was quantified using Qubit dsDNA BR/HS Assay Kit (Thermo Fisher) and normalized to 30ng input for library preparation. The first step PCR was performed in 15µl reactions including KAPA HiFi HotStart ReadyMix (Roche), 515F and 806R primers (Caporaso et al. 2011) (~350 bp fragment of the 16S V4 region) and template DNA (PCR program: 95°C for 3 min, 28x (98°C for 20 sec, 55°C for 15 sec, 72° for 15 sec), 72°C for 5 min). First PCR products were purified using 12µl AMPure XP beads and eluted in 26µL 10mM Tris-HCl. Indexing was performed in the second step PCR including KAPA HiFi HotStart ReadyMix (Roche), index primer mix (IDT for Illumina DNA/RNA UD Indexes, Tagmentation), purified first PCR product as template (PCR program: 95°C for 3 min, 8x (95°C for 30 sec, 55°C for 30 sec, 72°C for 30 sec), 72°C for 5 min). After another bead purification (14µl AMPure XP beads, eluted in 15µL 10mM Tris-HCl) the libraries were checked for correct fragment length on agarose gels, quantified using QuantiFluor dsDNA System (Promega) and pooled equimolarly. The pools were sequenced on an Illumina MiSeq device with v2 sequencing kits with 2 x 250 bp read length and a depth of 2–156k reads per sample (8 samples <10k reads/sample, 9 samples >10k & <20k reads/sample, average 69k reads/sample).

3.3. 16S rRNA gene amplicon analysis

Data processing, including quality control, reconstruction of sequences, taxonomic annotation, and diversity analysis was done using nf-core/ampliseq version 2.7.0 (doi: 10.5281/zenodo.10022435) [5] of the nf-core collection of workflows [6], utilising reproducible software environments from the Bioconda [7] and Biocontainers [8] projects.

The pipeline was executed with Nextflow v23.10.0 [9] and singularity v3.8.7 [10] with the following command: „`NXF_VER=23.10.0 nextflow run nf-core/ampliseq -r 2.7.0 -profile cfc --input samplesheet.tsv --FW_primer GTGYCAGCMGCCGCGGTAA --RV_primer GGACTACNVGGGTWTCTAAT --sample_inference pooled --trunclenf 200 --trunclenr 150 --min_len_asv 250 --max_len_asv 255 --cutadapt_min_overlap 15 --dada_ref_taxonomy silva=138 --skip_ancom --metadata metadata.tsv --outdir results`". Data quality was evaluated with FastQC v0.12.1 [11] and summarized with MultiQC v1.15 [12]. Cutadapt v3.4 [13] trimmed primers and all untrimmed sequences were discarded, because sequences that did not contain primer sequences were considered artifacts. Less than 7.5% of the sequences were discarded per sample and a mean of 96.9% of the sequences per sample passed the filtering. Adapter and primer-free sequences were processed as one pool with DADA2 v1.28.0 [14] to eliminate PhiX contamination, trim reads (forward reads at 200 bp and reverse reads at 150 bp, reads shorter than this were discarded), discard reads with > 2 expected errors, correct errors, merge read pairs, and remove polymerase chain reaction (PCR) chimeras. Ultimately,

9057 amplicon sequencing variants (ASVs) were obtained across all samples. The ASV count table contained in total 27,253,674 counts, at least 2,043 and at most 140,951 per sample (average 60,564). To remove spurious sequences, 7 ASVs with length lower than 250 or above 255 bp were removed with less than 0.42% counts per sample (9050 ASVs passed). Taxonomic classification was performed by DADA2 and the database 'Silva 138.1 prokaryotic SSU' [15]. ASV sequences, abundance and DADA2 taxonomic assignments were loaded into QIIME2 v2023.7.0 [16]. 19 ASVs that were annotated as mitochondria were discarded (9,031 ASVs passed). Within QIIME2, the final microbial community data was investigated for alpha (within-sample) and beta (between-sample) diversity after rarefaction to 2,042 counts.

3.4. Metabolome analysis

The metabolomics panel was chosen based on literature that involved physical activity, gut health, or microbial products; particularly short-chain fatty acids (SCFA), amino acids, bile acid derivates, choline metabolites, indole derivates, phenolic derivates, polyamines, and vitamins [17–28]. The panel encompassed Acetic acid, Butyric acid, Hexanoic acid, Indole, Isobutyric acid, Pentanoic acid, Pentanoic acid-4-methyl, Propanoic acid, Folate, Allantoin, IAA, Cholic acid, Deoxycholic acid, Lithocholic acid, 4-Hydroxyphenylacetic Acid, Salicylic acid, 3-Hydroxybenzoic acid, 4-Hydroxybenzoic acid, Enterolactone, Enterodiole, Tryptamine, Riboflavin, Niacin, Pyridoxine, Pantothenic acid, Putrescine, Spermidine, Spermine, Methionine, Leucine, Glutamate, Threonine, Creatinine, and Choline. However, Putrescine, Spermidine, Folate, and Pyridoxine were detected in less than 15% of samples and therefore omitted from further considerations.

Metabolomics sample preparation and measurements were performed at The Center for Plant Molecular Biology (ZMBP) of the University of Tübingen. Frozen samples were thawed and fully transferred to 5 mL reaction tubes. Tara weights and sample weights were documented prior to subsequent processing. Samples were then centrifuged using a Hettich swing out centrifuge for 10 minutes at 4500 rpm. The supernatant was transferred to new 2 mL reaction tubes and 1 ml was prepared for LC-MS, while the remaining volume was used for GC-MS. Resulting pellets were left for 20 minutes in the fume hood, then frozen at -80°C before freeze drying for 48 hours.

The LC-MS aliquot was dried using a speed vac, and pellet was resuspended using 200µL H_2O, 20% Methanol, 0.1% formic acid, with 9µM Lenk as internal standard. Samples were subjected to ultrasound for 10 minutes, 10 minutes incubation at room temperature, vortexing and again 10 minutes incubation at room temperature. Subsequently, samples were

centrifuged at 14000 rpm for 15 minutes at 4°C, and 100µL supernatants were transferred to autosampler and 3 µL were injected into LC-MS.

LC-MS analyses were carried out with a “Waters Acquity-SynaptG2” LC-MS system, operated at MS/MSE mode in parallel with a resolution of 10000, a scan time of 0.2 seconds and a scan range from 50 - 2000 m/z. For analyte separation an “Acquity UPLC HSS C18 SB, 2.1x100mm, 1.8µm” column was used with a 10 min gradient from Water with 0.1% Formic Acid (Solution A) to Methanol with 0.1% Formic Acid (Solution B).

GC-MS aliquots were centrifuged at 13000 rpm for 10 minutes at 4°C. Supernatant was transferred to 10 mL Headspace screwcap vials according to pipetting scheme Suppl. Table 5. Samples were measured on Shimadzu TQ8040 equipped with headspace sampler HS20, running in Headspace LOOP mode, settings are detailed in Supplemental File 2). Column Stabilwax-DA (Restek), length 30 m, diameter 0.32 mm, film thickness 1.0 µm was used. Peaks were checked manually and peak integration was performed using Labsolutions Insight GC/MS, while relative peak areas and metabolite amounts were calculated using R. Data was normalized using internal standards and stool concentration, calculated by the dry weight of stool in the total amount of stool sample given and all subsequent analyses were performed on normalized quantities of the metabolites.

Suppl. Table 5: Pipetting scheme for GC-MS samples. ISTD: Internal standard

Component	Volume [µL]
Saltout (contains 882 gL^{-1} of $(NH_4)_2SO_4$, and 238 gL^{-1} of NaH_2PO_4	3800
ISTD: 200 µM Butyric acid d8 (equals 20 nmol/Sample)	100
Sample	150
H_3PO_4	450
TOTAL	4500

4. Supplementary References

1. Breiman L, Friedman JH, Olshen RA, Stone CJ (2017) Classification And Regression Trees, 1st ed. Routledge

2. Bottiger LE (1973) Regular Decline in Physical Working Capacity with Age. BMJ 3:270–271. https://doi.org/10.1136/bmj.3.5874.270

3. Hawley JA, Noakes TD (1992) Peak power output predicts maximal oxygen uptake and performance time in trained cyclists. Eur J Appl Physiol 65:79–83. https://doi.org/10.1007/bf01466278

4. Storer TW, Davis JA, Caiozzo VJ (1990) Accurate prediction of &OV0312;O2max in cycle ergometry. Med Sci Sports Exerc 22:704–712. https://doi.org/10.1249/00005768-199010000-00024

5. Straub D, Blackwell N, Langarica-Fuentes A, Peltzer A, Nahnsen S, Kleindienst S (2020) Interpretations of Environmental Microbial Community Studies Are Biased by the Selected 16S rRNA (Gene) Amplicon Sequencing Pipeline. Front Microbiol 11:. https://doi.org/10.3389/fmicb.2020.550420

6. Ewels PA, Peltzer A, Fillinger S, Patel H, Alneberg J, Wilm A, Garcia MU, Di Tommaso P, Nahnsen S (2020) The nf-core framework for community-curated bioinformatics pipelines. Nat Biotechnol 38:276–278. https://doi.org/10.1038/s41587-020-0439-x

7. The Bioconda Team, Grüning B, Dale R, Sjödin A, Chapman BA, Rowe J, Tomkins-Tinch CH, Valieris R, Köster J (2018) Bioconda: sustainable and comprehensive software distribution for the life sciences. Nat Methods 15:475–476. https://doi.org/10.1038/s41592-018-0046-7

8. Da Veiga Leprevost F, Grüning BA, Alves Aflitos S, Röst HL, Uszkoreit J, Barsnes H, Vaudel M, Moreno P, Gatto L, Weber J, Bai M, Jimenez RC, Sachsenberg T, Pfeuffer J, Vera Alvarez R, Griss J, Nesvizhskii AI, Perez-Riverol Y (2017) BioContainers: an open-source and community-driven framework for software standardization. Bioinformatics 33:2580–2582. https://doi.org/10.1093/bioinformatics/btx192

9. Di Tommaso P, Chatzou M, Floden EW, Barja PP, Palumbo E, Notredame C (2017) Nextflow enables reproducible computational workflows. Nat Biotechnol 35:316–319. https://doi.org/10.1038/nbt.3820

10. Kurtzer GM, Sochat V, Bauer MW (2017) Singularity: Scientific containers for mobility of compute. PLOS ONE 12:e0177459. https://doi.org/10.1371/journal.pone.0177459

11. Andrews S (2010) FastQC: A Quality Control Tool for High Throughput Sequence Data

12. Ewels P, Magnusson M, Lundin S, Käller M (2016) MultiQC: summarize analysis results for multiple tools and samples in a single report. Bioinformatics 32:3047–3048. https://doi.org/10.1093/bioinformatics/btw354

13. Martin M (2011) Cutadapt removes adapter sequences from high-throughput sequencing reads. EMBnet.journal 17:10. https://doi.org/10.14806/ej.17.1.200

14. Callahan BJ, McMurdie PJ, Rosen MJ, Han AW, Johnson AJA, Holmes SP (2016) DADA2: High-resolution sample inference from Illumina amplicon data. Nat Methods 13:581–583. https://doi.org/10.1038/nmeth.3869

15. Quast C, Pruesse E, Yilmaz P, Gerken J, Schweer T, Yarza P, Peplies J, Glöckner FO (2012) The SILVA ribosomal RNA gene database project: improved data processing and web-based tools. Nucleic Acids Res 41:D590–D596. https://doi.org/10.1093/nar/gks1219

16. Bolyen E, Rideout JR, Dillon MR, Bokulich NA, Abnet CC, Al-Ghalith GA, Alexander H, Alm EJ, Arumugam M, Asnicar F, Bai Y, Bisanz JE, Bittinger K, Brejnrod A, Brislawn

CJ, Brown CT, Callahan BJ, Caraballo-Rodríguez AM, Chase J, Cope EK, Da Silva R, Diener C, Dorrestein PC, Douglas GM, Durall DM, Duvallet C, Edwardson CF, Ernst M, Estaki M, Fouquier J, Gauglitz JM, Gibbons SM, Gibson DL, Gonzalez A, Gorlick K, Guo J, Hillmann B, Holmes S, Holste H, Huttenhower C, Huttley GA, Janssen S, Jarmusch AK, Jiang L, Kaehler BD, Kang KB, Keefe CR, Keim P, Kelley ST, Knights D, Koester I, Kosciolek T, Kreps J, Langille MGI, Lee J, Ley R, Liu Y-X, Loftfield E, Lozupone C, Maher M, Marotz C, Martin BD, McDonald D, McIver LJ, Melnik AV, Metcalf JL, Morgan SC, Morton JT, Naimey AT, Navas-Molina JA, Nothias LF, Orchanian SB, Pearson T, Peoples SL, Petras D, Preuss ML, Pruesse E, Rasmussen LB, Rivers A, Robeson MS, Rosenthal P, Segata N, Shaffer M, Shiffer A, Sinha R, Song SJ, Spear JR, Swafford AD, Thompson LR, Torres PJ, Trinh P, Tripathi A, Turnbaugh PJ, Ul-Hasan S, Van Der Hooft JJJ, Vargas F, Vázquez-Baeza Y, Vogtmann E, Von Hippel M, Walters W, Wan Y, Wang M, Warren J, Weber KC, Williamson CHD, Willis AD, Xu ZZ, Zaneveld JR, Zhang Y, Zhu Q, Knight R, Caporaso JG (2019) Reproducible, interactive, scalable and extensible microbiome data science using QIIME 2. Nat Biotechnol 37:852–857. https://doi.org/10.1038/s41587-019-0209-9

17. Guzior DV, Quinn RA (2021) Review: microbial transformations of human bile acids. Microbiome 9:140. https://doi.org/10.1186/s40168-021-01101-1

18. Sarafian MH, Lewis MR, Pechlivanis A, Ralphs S, McPhail MJW, Patel VC, Dumas M-E, Holmes E, Nicholson JK (2015) Bile Acid Profiling and Quantification in Biofluids Using Ultra-Performance Liquid Chromatography Tandem Mass Spectrometry. Anal Chem 87:9662–9670. https://doi.org/10.1021/acs.analchem.5b01556

19. Courillon F, Gerhardt MF, Myara A, Rocchiccioli F, Trivin F (1997) The Optimized Use of Gas Chromatography-Mass Spectrometry and High Performance Liquid Chromatography to Analyse the Serum Bile Acids of Patients with Metabolic Cholestasis and Peroxisomal Disorders. Clin Chem Lab Med 35:. https://doi.org/10.1515/cclm.1997.35.12.919

20. Cui M, Trimigno A, Castro-Mejía JL, Reitelseder S, Bülow J, Bechshøft RL, Nielsen DS, Holm L, Engelsen SB, Khakimov B (2021) Human Fecal Metabolome Reflects Differences in Body Mass Index, Physical Fitness, and Blood Lipoproteins in Healthy Older Adults. Metabolites 11:717. https://doi.org/10.3390/metabo11110717

21. Shi Y, Wang P, Zhou D, Huang L, Zhang L, Gao X, Maitiabula G, Wang S, Wang X (2022) Multi-Omics Analyses Characterize the Gut Microbiome and Metabolome Signatures of Soldiers Under Sustained Military Training. Front Microbiol 13:827071. https://doi.org/10.3389/fmicb.2022.827071

22. Gao K, Mu C, Farzi A, Zhu W (2020) Tryptophan Metabolism: A Link Between the Gut Microbiota and Brain. Adv Nutr 11:709–723. https://doi.org/10.1093/advances/nmz127

23. Wohlfarth A, Weinmann W, Dresen S (2010) LC-MS/MS screening method for designer amphetamines, tryptamines, and piperazines in serum. Anal Bioanal Chem 396:2403–2414. https://doi.org/10.1007/s00216-009-3394-4

24. Pautova A, Khesina Z, Getsina M, Sobolev P, Revelsky A, Beloborodova N (2020) Determination of Tryptophan Metabolites in Serum and Cerebrospinal Fluid Samples Using Microextraction by Packed Sorbent, Silylation and GC–MS Detection. Molecules 25:3258. https://doi.org/10.3390/molecules25143258

25. O’Donovan CM, Madigan SM, Garcia-Perez I, Rankin A, O’ Sullivan O, Cotter PD (2020) Distinct microbiome composition and metabolome exists across subgroups of

elite Irish athletes. J Sci Med Sport 23:63–68. https://doi.org/10.1016/j.jsams.2019.08.290

26. Goucher E, Kicman A, Wolff K, Smith N, Jickells S (2010) Hydrophilic stationary phases: A practical approach for the co-analysis of compounds with varying polarity in biological matrices. J Sep Sci 33:955–965. https://doi.org/10.1002/jssc.200900727

27. Kand'ár R, Žáková P (2008) Allantoin as a marker of oxidative stress in human erythrocytes. Clin Chem Lab Med 46:. https://doi.org/10.1515/CCLM.2008.244

28. García-Villalba R, Giménez-Bastida JA, García-Conesa MT, Tomás-Barberán FA, Carlos Espín J, Larrosa M (2012) Alternative method for gas chromatography-mass spectrometry analysis of short-chain fatty acids in faecal samples. J Sep Sci 35:1906–1913. https://doi.org/10.1002/jssc.201101121
