# Supplementary material for: Resistance Training Reshapes the Gut Microbiome in a Longitudinal 8-Week Intervention in Sedentary Adults
Source: Sports Med Open. 2026 Mar 16;12:21. doi: 10.1186/s40798-026-00990-6 (PMC12989468; doi:10.1186/s40798-026-00990-6)
Supplement: Supplementary file 2 — Supplementary Material 2. [file 40798_2026_990_MOESM2_ESM.pdf]

Datenblatt Screening

**Einschlusskriterien:**	JA	NEIN
25-60 Jahre alt	☐	☐
BMI zwischen 18,5- 30	☐	☐
Weniger als 1hr regelmäßige körperliche sportliche Aktivität pro Woche/letzten 6 Monaten	☐	☐
Unterschriebene Einwilligungserklärung	☐	☐
**Ausschlusskriterien:**	Nein	JA
Morbus Crohn	☐	☐
Colitis Ulcerosa	☐	☐
Reizdarmsyndrom	☐	☐
Gluten Sensitivität	☐	☐
Diabetes Typ 1 oder 2	☐	☐
Krebserkrankung ohne/mit Chemotherapie im Zeitraum < 1 Jahr	☐	☐
Körperliche oder gesundheitliche Einschränkungen, die das sportliche Training verhindern	☐	☐
Schwangerschaft, Stillend, Geburt < 6 Monate	☐	☐
Antibiotika bis zu 6 Monate vor Studienbeginn	☐	☐
Einnahme von Protonenpumpen-Inhibitoren (PPI)	☐	☐
Andere Studienteilnahme innerhalb der letzten 2 Monate	☐	☐
Nicht Einwilligungsfähige Studienteilnehmer	☐	☐

Teilnehmer erfüllt Teilnahmebedingungen für diese Studie: ☐ JA ☐ NEIN

Unterschrift Aufklärender Trainer Datum (tt.mm.jjjj)

Randomisiert in folgende Trainingsgruppe: ☐ Endurance

☐ Strength

Terminplan

Terminvergabe	Datum<br>(tt.mm.jjjj)	Uhrzeit<br>(hh:mm)
**Screening**		
**Baseline**<br>▪ **Fitnesstest und Einweisung**		
**4 Wochen**<br>▪ **Fitnesstest**		
**8 Wochen**<br>▪ **Fitnesstest und Abschluss**		

Einweisender Trainer: ____

Trainingsplan wöchentliche Termine

	Woche 1	Woche 2	Woche 3	Woche 4	Woche 5	Woche 6	Woche 7	Woche 8
**Datum**<br>**Uhrzeit**	= Baseline Termin							
Absolviert	☐	☐	☐	☐	☐	☐	☐	☐
**Datum**<br>**Uhrzeit**								
Absolviert	☐	☐	☐	☐	☐	☐	☐	☐
**Datum**<br>**Uhrzeit**				= 4 Wochen Termin				= 8 Wochen Termin
Absolviert	☐	☐	☐	☐	☐	☐	☐	☐

Hinweis:
Der Teilnehmer sollte nach seinen Urlaubsplänen gefragt werden.
Sollte der Teilnehmer innerhalb der Studiendurchführung einen längeren Urlaub (> 5 Tage) geplant haben, ist ein Start erst nach dem Urlaub möglich!!!

Fragebogen: Demographische Daten

Datum: _____._____.________

Geburtsjahr: __ __ __ __ (jjjj) **Geschlecht:** ☐ Mann ☐ Frau

Größe: __ __ __ cm **Gewicht:** __ __ __ kg

Geburtsland: __________________________ **Ständiger Aufenthalt in Deutschland seit:** __ __ __ __ (jjjj)

- **Waren Sie am CORONA-Virus erkrankt?** ☐ Nein ☐ Ja
  - **Symptome: Start:** __ __ / __ __ __ __(mm/jjjj) **Ende**: __ __ / __ __ __ __(mm/jjjj)
  - **Aktuell Symptome?** ☐ Nein ☐ Ja: welche?____
  - **Verlauf?** ☐ ohne Krankenhausaufenthalt
    ☐ mit Krankenhausaufenthalt ohne intensivmedizinische Betreuung
    ☐ mit Krankenhausaufenthalt mit intensivmedizinischer Betreuung

  ➔ **Wurden Sie geimpft?** ☐ Nein ☐ Ja:
  1. Impfung Wann: __ __ / __ __ __ __(mm/jjjj) Impfstoff: ☐ Moderna ☐ Biontech ☐ Astrazeneca ☐ _______________
  2. Impfung Wann: __ __ / __ __ __ __(mm/jjjj) Impfstoff:☐ Moderna ☐ Biontech ☐ Astrazeneca ☐ _______________
  Booster Impfung : Wann: __ __ / __ __ __ __(mm/jjjj) Impfstoff:☐ Moderna ☐ Biontech ☐ ______________

- **Haben Sie in den letzten 2 Jahren auffällig viel Gewicht verloren oder zugenommen?** ☐ Nein ☐ Ja:
  Ausgangsgewicht? ___ ___ ___kg Wieviel ab/zugenommen? ___ ___ ___ kg
  ➔ **Welchen Grund gab es hierfür?** ☐ Erkrankung ☐ Medikamentöse Therapie ☐ Diät
  ☐ Sport/Training ☐ unklar ☐ Anderer: ____

- **Haben Sie in den letzten 6 Monaten eine Diät durchgeführt:** ☐ Nein ☐ Ja:
  ☐ aktuell ☐ innerhalb der letzten 6 Wochen ☐ vor mehr als 6 Wochen
  ➔ Welche Diät Form? ____

- **Sind Sie in den letzten 10 Jahren min. 1 Jahr lang (3x/Woche) sportlich aktiv gewesen?** ☐ Nein ☐ Ja:

**Sportart**	**Zeitraum (jjjj-jjjj)**	**Fokus auf:**
		☐ Wettkampfsport ☐ Freizeitsport
		☐ Wettkampfsport ☐ Freizeitsport
		☐ Wettkampfsport ☐ Freizeitsport

- **Wie sieht Ihr aktuelles alltägliches Bewegungsprofil aus?**

**Bewegungsart** (spazieren, radeln (E-Bike), etc.)	**Distanz in km**	**Häufigkeit**
		☐Täglich ☐1-2x Woche ☐3-4x Woche
		☐Täglich ☐1-2x Woche ☐3-4x Woche
		☐Täglich ☐1-2x Woche ☐3-4x Woche

- **Allergien/ Unverträglichkeiten:** ☐ Nein ☐ Ja:
  ☐ Lactose ☐ Gluten ☐ Heuschnupfen ☐ andere:____

- **Wurde bei Ihnen schon einmal eine Krebsdiagnose gestellt?** ☐ Nein ☐ Ja:
  ➔ Welche: _____________________________________ Wann: ___________________(jjjj)

- **Haben Sie eine Chemotherapie erhalten:** ☐ Nein ☐ Ja:
  ☐ Vor mehr als 1 Jahr ☐ vor mehr als 2 Jahre ☐ vor mehr als 3 Jahren

- **Haben Sie Immunsuppressiva eingenommen:** ☐ Nein ☐ Ja:
  ☐ Vor mehr als 1 Jahr ☐ vor mehr als 6 Monate ☐ vor mehr als 3 Monaten
  ☐ vor mehr als 4 Wochen ☐ vor max. 4 Wochen ☐ aktuell

- **Wann haben Sie das letzte Mal Antibiotika eingenommen?** __________________(mm/jjjj)

- **Hatten Sie in den letzten 4 Wochen folgende Symptome?**
  ☐ Durchfall ☐ Verstopfung ☐ Grippaler Infekt ☐ Fieber ☐ Andere:__________________________

Teilnehmer Nr.: **100**

- **Leiden Sie aktuell unter Erkrankungen/Verletzungen?** ☐ Nein ☐ Ja:

**Medizinische Diagnose** z.B. Bluthochdruck/ Asthma/ Sprunggelenkszerrung/ OPs	**Start Datum** (mm/jjjj)	**Nehmen sie deswegen Medikamente?** (Name)
		☐ Nein ☐ Ja:
		☐ Nein ☐ Ja:
		☐ Nein ☐ Ja:

- **Nehmen Sie aktuell regelmäßig Medikamente ein?** ☐ Nein ☐ Ja:

**Name**	**Dosierung**	**Einheit** (mg, g)	**Verabreichungsart** (oral, subcutan,…)	**Grund der Einnahme**	**Start** (mm/jjjj)
z.b. Aspirin	1x tgl.	100mg	Oral	Bluthochdruck	10/1999

- **Haben Sie in den letzten 6 Monaten Abführmittel eingenommen?** ☐ Nein ☐ Ja:

**Name**	**Einnahmeart** (Tropfen, Tabletten)	**Häufigkeit**
		☐ 1x/Monat ☐ 2-3x/Monat<br>☐ 1-2x/Woche ☐ täglich
		☐ 1x/Monat ☐ 2-3x/Monat<br>☐ 1-2x/Woche ☐ täglich

- **Nehmen Sie aktuell regelmäßig Nahrungsergänzungsmittel ein?** ☐ Nein ☐ Ja:

**Name** z.B. Vitamine/Mineralstoffe/etc.	**Dosierung**	**Einheit** (mg, g)	**Verabreichungsart** (oral, i.v., …)	**Grund der Einnahme**	**Start** (mm/jjjj)

- **Wie sieht und sah Ihre Ernährungsform in den letzten 12 Monaten aus?**

**Ernährungsformen**	**Nein/Ja**	**Start (mm/jjjj)**	**Ende (mm/jjjj)**
**Keine spezielle Ernährungsform** (normal)	☐ Nein ☐ Ja	__ __/ __ __ __ __	__ __/ __ __ __ __
**Vegan** (keine tierischen Nahrungsmittel)	☐ Nein ☐ Ja	__ __/ __ __ __ __	__ __/ __ __ __ __
**Vegetarisch** (inkl. Fisch)	☐ Nein ☐ Ja	__ __/ __ __ __ __	__ __/ __ __ __ __
**Vegetarisch** (ohne Fisch)	☐ Nein ☐ Ja	__ __/ __ __ __ __	__ __/ __ __ __ __
**Glutenfrei**	☐ Nein ☐ Ja	__ __/ __ __ __ __	__ __/ __ __ __ __
**Laktosefrei**	☐ Nein ☐ Ja	__ __/ __ __ __ __	__ __/ __ __ __ __
**Low Carb**	☐ Nein ☐ Ja	__ __/ __ __ __ __	__ __/ __ __ __ __
**Low Fat**	☐ Nein ☐ Ja	__ __/ __ __ __ __	__ __/ __ __ __ __
**High Protein**	☐ Nein ☐ Ja	__ __/ __ __ __ __	__ __/ __ __ __ __
**Andere:**	☐ Nein ☐ Ja	__ __/ __ __ __ __	__ __/ __ __ __ __

Wie haben Sie von dieser Studie erfahren? ____

Vielen Dank für Ihre Teilnahme!

Fragebogen: Baseline

Datum: _____._____.________

Bitte füllen Sie diesen Fragebogen vor Ihrem Fitnesstest vollständig und in Ruhe aus. Sollten Unklarheiten bestehen können Sie sich jederzeit an Ihren Trainer wenden. Geben Sie den Fragebogen bitte anschließend bei Ihrem Trainer ab. Vielen Dank!

- **Hat sich an Ihrer Medikation seit dem Screening Termin etwas verändert?** ☐ Nein ☐ Ja:

**Name**	**Dosierung**	**Einheit** (mg, g)	**Verabreichungsart** (oral, subcutan,…)	**Grund der Einnahme**	**Start / Ende** (mm/jjjj)

- **Hat sich an Ihrer Ernährungsform/ Diät seit dem Screening Termin etwas verändert?** ☐ Nein ☐ Ja:
  (z.B. low carb, vegan,…)

**Ernährungsform eintragen**	**Datum Beginn (mm/jjjj)**	**Datum Ende (mm/jjjj)**
	__ __/__ __ __ __	__ __/__ __ __ __
	__ __/__ __ __ __	__ __/__ __ __ __

- **Rauchen Sie oder haben Sie geraucht?** ☐ Nein ☐ Ja:
  ☐ Ex-Raucher -> Aufgehört seit (ungefähr): ______________(mm.jjjj)
  ☐ Aktiver Raucher-> Seit (ungefähr): _____________(mm.jjjj)

Zigaretten/Tabak	☐ Nein ☐ Ja	_____ Stück/Tag	_____ Stück/Woche
E-Zigaretten	☐ Nein ☐ Ja	_____ Stück/Tag	_____ Stück/Woche
Andere:_____________	☐ Nein ☐ Ja	_____ Stück/Tag	_____ Stück/Woche

- **Was/ Wieviel trinken Sie aktuell über den Tag verteilt?**

Wasser/ ungesüßte Tees	☐ nie / selten ☐ <1l/Tag ☐ 1-2l/Tag ☐ >3l/Tag
Kaffee	☐ nie / selten ☐ <1 Tasse/Tag ☐ 2-3 Tassen/Tag ☐ >4 Tassen/Tag
Säfte/ Limonaden	☐ nie / selten ☐ <1l/Tag ☐ 1-2l/Tag ☐ >3l/Tag
____________________________	☐ nie / selten ☐ <1l/Tag ☐ 1-2l/Tag ☐ >3l/Tag

- **Haben Sie in den letzten 4 Wochen Alkohol getrunken?** ☐ Nein ☐ Ja:

**Bier** (0,33l= 1 Flache)	**Wein** (0,2l = 1 Glas)	**Spirituosen** (0,02l = 1 Glas)	**Andere:** (0,2l = 1 Glas)
☐ Nein<br>☐ Ja: ____Flaschen/Woche	☐ Nein<br>☐ Ja:____Gläser /Woche	☐ Nein<br>☐ Ja:____ Gläser/Woche	☐ Nein<br>☐ Ja:____Gläser /Woche

- **Haben Sie in den letzten 4 Wochen Prä-/Probiotika zu sich genommen?** ☐ Nein ☐ Ja:
  ☐ gelegentlich (1-2x/ Monat) ☐ regelmäßig (min. 1-2x/ Woche) ☐ täglich

- **Wie sah Ihre Ernährung im Detail in den letzten 4 Wochen aus?**

**Gemüse / Obst**	☐ Nie<br>☐ Weniger als 1x pro Woche<br>☐ 1-2x pro Woche<br>☐ 3-4x pro Woche	☐ 4-6x pro Woche<br>☐ täglich<br>☐ mehrmals täglich
**Fisch**	☐ Nie<br>☐ Weniger als 1x pro Woche<br>☐ 1-2x pro Woche<br>☐ 3-4x pro Woche	☐ 4-6x pro Woche<br>☐ täglich<br>☐ mehrmals täglich
**Fleisch**	☐ Nie<br>☐ Weniger als 1x pro Woche<br>☐ 1-2x pro Woche<br>☐ 3-4x pro Woche	☐ 4-6x pro Woche<br>☐ täglich<br>☐ mehrmals täglich
**Eier**	☐ Nie<br>☐ Weniger als 1x pro Woche<br>☐ 1-2x pro Woche<br>☐ 3-4x pro Woche	☐ 4-6x pro Woche<br>☐ täglich<br>☐ mehrmals täglich
**Milchprodukte**	☐ Nie<br>☐ Weniger als 1x pro Woche<br>☐ 1-2x pro Woche<br>☐ 3-4x pro Woche	☐ 4-6x pro Woche<br>☐ täglich<br>☐ mehrmals täglich
**Getreideprodukte**	☐ Nie<br>☐ Weniger als 1x pro Woche<br>☐ 1-2x pro Woche<br>☐ 3-4x pro Woche	☐ 4-6x pro Woche<br>☐ täglich<br>☐ mehrmals täglich
**Süßigkeiten**<br>(Schokolade, Gummibärchen, Eis, Kuchen, etc.)	☐ Nie<br>☐ Weniger als 1x pro Woche<br>☐ 1-2x pro Woche<br>☐ 3-4x pro Woche	☐ 4-6x pro Woche<br>☐ täglich<br>☐ mehrmals täglich
**Salzige Snacks**<br>(Chips, Salzstangen, geröstete Erdnüsse, etc.)	☐ Nie<br>☐ Weniger als 1x pro Woche<br>☐ 1-2x pro Woche<br>☐ 3-4x pro Woche	☐ 4-6x pro Woche<br>☐ täglich<br>☐ mehrmals täglich

Fitnesstest: Baseline

Datum: _____._____.________

Der Teilnehmer sollte vor dem Fitnesstest zur Ruhe kommen und 10-15 Minuten sitzen. In dieser Zeit kann z.B. der Fragebogen ausgefüllt werden. Der Fitnesstest erfolgt immer am selben Gerät mit den gleichen Voreinstellungen (Crosstrainer).

Der Teilnehmer ist randomisiert in: ☐ Endurance

☐ Strength

Ruhe Puls: ___ ___ ___ Herzschläge/Minute	Ruhe Blutdruck:___ ___ ___/___ ___ ___mmHg
Körperfett: ____%	BMI:__ __ , __ __ kg/m²
VO2max: _________________ml/kg/min	
**Wurde der Fitnesstest erfolgreich durchgeführt? ☐ Ja ☐ Nein:**	

Körperregion	Biologisches Alter	Geräte Übung	Kraft in Kg
**Oberkörper**	_____Jahre	Ruderzug	__ __ __,__ kg
		Latzug	__ __ __,__ kg
		Brustpresse	__ __ __,__ kg
**Rumpf**	_____Jahre	Rückentrainer	__ __ __,__ kg
		Bauchtrainer	__ __ __,__ kg
**Beine**	_____Jahre	Beinbeuger	__ __ __,__ kg
		Beinpresse	__ __ __,__ kg
**Biologisches Alter Gesamt**	_____Jahre	Kommentar:	

Stuhlproben Tracking	Datum (tt.mm.jjjj)	Probe ok?
**Entnahme durch Teilnehmer**		☐ Ja ☐ Nein:
**Abgabe von 2 Proben im Mapet**		

Name Trainer **Unterschrift** **Datum (tt.mm.jjjj)**

Fragebogen: 4 Wochen

Datum: _____._____.________

Bitte Bitte füllen Sie diesen Fragebogen vor Ihrem Fitnesstest vollständig und in Ruhe aus. Sollten Unklarheiten bestehen können Sie sich jederzeit an Ihren Trainer wenden. Geben Sie den Fragebogen bitte anschließend bei Ihrem Trainer ab. Vielen Dank!

- **Hat sich an Ihrer Medikation seit dem Baseline Termin etwas verändert?** ☐ Nein ☐ Ja:

**Name**	**Dosierung**	**Einheit** (mg, g)	**Verabreichungsart** (oral, subcutan,…)	**Grund der Einnahme**	**Start / Ende** (mm/jjjj)

- **Hat sich an Ihrer Ernährungsform/ Diät seit dem Baseline Termin etwas verändert?** ☐ Nein ☐ Ja:
  (z.B. low carb, vegan,…)

**Ernährungsform eintragen**	**Datum Beginn (mm/jjjj)**	**Datum Ende (mm/jjjj)**
	__ __/__ __ __ __	__ __/__ __ __ __
	__ __/__ __ __ __	__ __/__ __ __ __

- **Rauchen Sie oder haben Sie geraucht?** ☐ Nein ☐ Ja:
  ☐ Ex-Raucher -> Aufgehört seit (ungefähr): ______________(mm.jjjj)
  ☐ Aktiver Raucher-> Seit (ungefähr): _____________(mm.jjjj)

Zigaretten/Tabak	☐ Nein ☐ Ja	_____ Stück/Tag	_____ Stück/Woche
E-Zigaretten	☐ Nein ☐ Ja	_____ Stück/Tag	_____ Stück/Woche
Andere:_____________	☐ Nein ☐ Ja	_____ Stück/Tag	_____ Stück/Woche

- **Was/ Wieviel trinken Sie aktuell über den Tag verteilt?**

Wasser/ ungesüßte Tees	☐ nie / selten ☐ <1l/Tag ☐ 1-2l/Tag ☐ >3l/Tag
Kaffee	☐ nie / selten ☐ <1 Tasse/Tag ☐ 2-3 Tassen/Tag ☐ >4 Tassen/Tag
Säfte/ Limonaden	☐ nie / selten ☐ <1l/Tag ☐ 1-2l/Tag ☐ >3l/Tag
___________________________	☐ nie / selten ☐ <1l/Tag ☐ 1-2l/Tag ☐ >3l/Tag

- **Haben Sie in den letzten 4 Wochen Alkohol getrunken?** ☐ Nein ☐ Ja:

**Bier** (0,33l= 1 Flache)	**Wein** (0,2l = 1 Glas)	**Spirituosen** (0,02l = 1 Glas)	**Andere:** (0,2l = 1 Glas)
☐ Nein<br>☐ Ja: ____Flaschen/Woche	☐ Nein<br>☐ Ja:____Gläser /Woche	☐ Nein<br>☐ Ja:____ Gläser/Woche	☐ Nein<br>☐ Ja:____Gläser /Woche

- **Haben Sie in den letzten 4 Wochen Prä-/Probiotika zu sich genommen?** ☐ Nein ☐ Ja:

☐ gelegentlich (1-2x/ Monat) ☐ regelmäßig (min. 1-2x/ Woche) ☐ täglich

- **Wie sah Ihre Ernährung im Detail in den letzten 4 Wochen aus?**

**Gemüse / Obst**	☐ Nie<br>☐ Weniger als 1x pro Woche<br>☐ 1-2x pro Woche<br>☐ 3-4x pro Woche	☐ 4-6x pro Woche<br>☐ täglich<br>☐ mehrmals täglich
**Fisch**	☐ Nie<br>☐ Weniger als 1x pro Woche<br>☐ 1-2x pro Woche<br>☐ 3-4x pro Woche	☐ 4-6x pro Woche<br>☐ täglich<br>☐ mehrmals täglich
**Fleisch**	☐ Nie<br>☐ Weniger als 1x pro Woche<br>☐ 1-2x pro Woche<br>☐ 3-4x pro Woche	☐ 4-6x pro Woche<br>☐ täglich<br>☐ mehrmals täglich
**Eier**	☐ Nie<br>☐ Weniger als 1x pro Woche<br>☐ 1-2x pro Woche<br>☐ 3-4x pro Woche	☐ 4-6x pro Woche<br>☐ täglich<br>☐ mehrmals täglich
**Milchprodukte**	☐ Nie<br>☐ Weniger als 1x pro Woche<br>☐ 1-2x pro Woche<br>☐ 3-4x pro Woche	☐ 4-6x pro Woche<br>☐ täglich<br>☐ mehrmals täglich
**Getreideprodukte**	☐ Nie<br>☐ Weniger als 1x pro Woche<br>☐ 1-2x pro Woche<br>☐ 3-4x pro Woche	☐ 4-6x pro Woche<br>☐ täglich<br>☐ mehrmals täglich
**Süßigkeiten**<br>(Schokolade, Gummibärchen, Eis, Kuchen, etc.)	☐ Nie<br>☐ Weniger als 1x pro Woche<br>☐ 1-2x pro Woche<br>☐ 3-4x pro Woche	☐ 4-6x pro Woche<br>☐ täglich<br>☐ mehrmals täglich
**Salzige Snacks**<br>(Chips, Salzstangen, geröstete Erdnüsse, etc.)	☐ Nie<br>☐ Weniger als 1x pro Woche<br>☐ 1-2x pro Woche<br>☐ 3-4x pro Woche	☐ 4-6x pro Woche<br>☐ täglich<br>☐ mehrmals täglich

Fitnesstest: 4 Wochen

Datum: _____._____.________

Der Teilnehmer sollte vor dem Fitnesstest zur Ruhe kommen und 10-15 Minuten sitzen. In dieser Zeit kann z.B. der Fragebogen ausgefüllt werden. Der Fitnesstest erfolgt immer am selben Gerät mit den gleichen Voreinstellungen (Crosstrainer).

Der Teilnehmer ist randomisiert in: ☐ Endurance

☐ Strength

Ruhe Puls: ___ ___ ___ Herzschläge/Minute	Ruhe Blutdruck:___ ___ ___/___ ___ ___mmHg
Körperfett: ____%	BMI:__ __ , __ __ kg/m²
VO2max: _________________ml/kg/min	
**Wurde der Fitnesstest erfolgreich durchgeführt? ☐ Ja ☐ Nein:**	

Körperregion	Biologisches Alter/Körperregion	Geräte Übung	Kraft in Kg
**Oberkörper**	_____Jahre	Ruderzug	__ __ __,__ kg
		Latzug	__ __ __,__ kg
		Brustpresse	__ __ __,__ kg
**Rumpf**	_____Jahre	Rückentrainer	__ __ __,__ kg
		Bauchtrainer	__ __ __,__ kg
**Beine**	_____Jahre	Beinbeuger	__ __ __,__ kg
		Beinpresse	__ __ __,__ kg
**Biologisches Alter Gesamt**	_____Jahre	Kommentar:	

Stuhlproben Tracking	Datum (tt.mm.jjjj)	Probe ok?
**Entnahme durch Teilnehmer**		☐ Ja ☐ Nein:
**Abgabe von 1 Probe im Mapet**		

Name Trainer **Unterschrift** **Datum (tt.mm.jjjj)**

Fragebogen: 8 Wochen

Datum: _____._____._________

Bitte füllen Sie diesen Fragebogen vor Ihrem Fitnesstest vollständig und in Ruhe aus. Sollten Unklarheiten bestehen können Sie sich jederzeit an Ihren Trainer wenden. Geben Sie den Fragebogen bitte anschließend bei Ihrem Trainer ab. Vielen Dank!

- **Hat sich an Ihrer Medikation seit dem 4 Wochen Termin etwas verändert?** ☐ Nein ☐ Ja:

**Name**	**Dosierung**	**Einheit** (mg, g)	**Verabreichungsart** (oral, subcutan,…)	**Grund der Einnahme**	**Start / Ende** (mm/jjjj)

- **Hat sich an Ihrer Ernährungsform/ Diät seit dem 4 Wochen Termin etwas verändert?** ☐ Nein ☐ Ja:
  (z.B. low carb, vegan,…)

**Ernährungsform eintragen**	**Datum Beginn (mm/jjjj)**	**Datum Ende (mm/jjjj)**
	__ __/__ __ __ __	__ __/__ __ __ __
	__ __/__ __ __ __	__ __/__ __ __ __

- **Rauchen Sie oder haben Sie geraucht?** ☐ Nein ☐ Ja:
  ☐ Ex-Raucher -> Aufgehört seit (ungefähr): ______________(mm.jjjj)
  ☐ Aktiver Raucher-> Seit (ungefähr): _____________(mm.jjjj)

Zigaretten/ Tabak	☐ Nein ☐ Ja	_____ Stück/Tag	_____ Stück/Woche
E-Zigaretten	☐ Nein ☐ Ja	_____ Stück/Tag	_____ Stück/Woche
Andere:_____________	☐ Nein ☐ Ja	_____ Stück/Tag	_____ Stück/Woche

- **Was/ Wieviel trinken Sie aktuell über den Tag verteilt?**

Wasser/ ungesüßte Tees	☐ nie / selten ☐ <1l/Tag ☐ 1-2l/Tag ☐ >3l/Tag
Kaffee	☐ nie / selten ☐ <1 Tasse/Tag ☐ 2-3 Tassen/Tag ☐ >4 Tassen/Tag
Säfte/ Limonaden	☐ nie / selten ☐ <1l/Tag ☐ 1-2l/Tag ☐ >3l/Tag
___________________________	☐ nie / selten ☐ <1l/Tag ☐ 1-2l/Tag ☐ >3l/Tag

- **Haben Sie in den letzten 4 Wochen Alkohol getrunken?** ☐ Nein ☐ Ja:

**Bier** (0,33l= 1 Flache)	**Wein** (0,2l = 1 Glas)	**Spirituosen** (0,02l = 1 Glas)	**Andere:** (0,2l = 1 Glas)
☐ Nein<br>☐ Ja: ____Flaschen/Woche	☐ Nein<br>☐ Ja:____Gläser /Woche	☐ Nein<br>☐ Ja:____ Gläser/Woche	☐ Nein<br>☐ Ja:____Gläser /Woche

- **Haben Sie in den letzten 4 Wochen Prä-/Probiotika zu sich genommen?** ☐ Nein ☐ Ja:
  ☐ gelegentlich (1-2x/ Monat) ☐ regelmäßig (min. 1-2x/ Woche) ☐ täglich

- **Wie sah Ihre Ernährung im Detail in den letzten 4 Wochen aus?**

**Gemüse / Obst**	☐ Nie<br>☐ Weniger als 1x pro Woche<br>☐ 1-2x pro Woche<br>☐ 3-4x pro Woche	☐ 4-6x pro Woche<br>☐ täglich<br>☐ mehrmals täglich
**Fisch**	☐ Nie<br>☐ Weniger als 1x pro Woche<br>☐ 1-2x pro Woche<br>☐ 3-4x pro Woche	☐ 4-6x pro Woche<br>☐ täglich<br>☐ mehrmals täglich
**Fleisch**	☐ Nie<br>☐ Weniger als 1x pro Woche<br>☐ 1-2x pro Woche<br>☐ 3-4x pro Woche	☐ 4-6x pro Woche<br>☐ täglich<br>☐ mehrmals täglich
**Eier**	☐ Nie<br>☐ Weniger als 1x pro Woche<br>☐ 1-2x pro Woche<br>☐ 3-4x pro Woche	☐ 4-6x pro Woche<br>☐ täglich<br>☐ mehrmals täglich
**Milchprodukte**	☐ Nie<br>☐ Weniger als 1x pro Woche<br>☐ 1-2x pro Woche<br>☐ 3-4x pro Woche	☐ 4-6x pro Woche<br>☐ täglich<br>☐ mehrmals täglich
**Getreideprodukte**	☐ Nie<br>☐ Weniger als 1x pro Woche<br>☐ 1-2x pro Woche<br>☐ 3-4x pro Woche	☐ 4-6x pro Woche<br>☐ täglich<br>☐ mehrmals täglich
**Süßigkeiten**<br>(Schokolade, Gummibärchen, Eis, Kuchen, etc.)	☐ Nie<br>☐ Weniger als 1x pro Woche<br>☐ 1-2x pro Woche<br>☐ 3-4x pro Woche	☐ 4-6x pro Woche<br>☐ täglich<br>☐ mehrmals täglich
**Salzige Snacks**<br>(Chips, Salzstangen, geröstete Erdnüsse, etc.)	☐ Nie<br>☐ Weniger als 1x pro Woche<br>☐ 1-2x pro Woche<br>☐ 3-4x pro Woche	☐ 4-6x pro Woche<br>☐ täglich<br>☐ mehrmals täglich

Fitnesstest: 8 Wochen

Datum: _____._____.________

Der Teilnehmer sollte vor dem Fitnesstest zur Ruhe kommen und 10-15 Minuten sitzen. In dieser Zeit kann z.B. der Fragebogen ausgefüllt werden. Der Fitnesstest erfolgt immer am selben Gerät mit den gleichen Voreinstellungen (Crosstrainer).

Der Teilnehmer ist randomisiert in: ☐ Endurance

☐ Strength

Ruhe Puls: ___ ___ ___ Herzschläge/Minute	Ruhe Blutdruck:___ ___ ___/___ ___ ___mmHg
Körperfett: ____%	BMI:__ __ , __ __ kg/m²
VO2max: _________________ml/kg/min	
**Wurde der Fitnesstest erfolgreich durchgeführt? ☐ Ja ☐ Nein:**	

Körperregion	Biologisches Alter/Körperregion	Geräte Übung	Kraft in Kg
**Oberkörper**	_____Jahre	Ruderzug	__ __ __,__ kg
		Latzug	__ __ __,__ kg
		Brustpresse	__ __ __,__ kg
**Rumpf**	_____Jahre	Rückentrainer	__ __ __,__ kg
		Bauchtrainer	__ __ __,__ kg
**Beine**	_____Jahre	Beinbeuger	__ __ __,__ kg
		Beinpresse	__ __ __,__ kg
**Biologisches Alter Gesamt**	_____Jahre	Kommentar:	

Stuhlproben Tracking	Datum (tt.mm.jjjj)	Probe ok?
**Entnahme durch Teilnehmer**		☐ Ja ☐ Nein:
**Abgabe von 2 Proben im Mapet**		

Name Trainer **Unterschrift** **Datum (tt.mm.jjjj)**

Finaler Status: Ende der Studie

❍ **Erfolgreich** (Teilnehmer hat die Studie erfolgreich abgeschlossen)
❍ **Wiederruf** vor Erreichen eines Endpunktes durch:

- ❑ Teilnehmer wiederruft die Teilnahme nach Unterschrift der Einwilligung
- ❑ Früher Ausschluss bedingt durch eine Protokoll Verletzung
  - Unsachgemäße Studiendurchführung
  - Mangelnde Compliance des Teilnehmers
  - Andere: ____________________________________
- ❑ Andere: ____

Seite vervollständigt durch: ____ (Vor/ Nachname)

Unterschrift: ____________________________ **Datum:** ______________________________ (tt.mm.jjjj)

Ende der Studie
Der Principal Investigator bestätigt die Korrektheit der erhobenen Daten in diesem Worksheet.

Name PI: ____ (Vor/ Nachname)

Unterschrift PI: __________________________ **Datum:** _____________________________ (tt.mm.jjjj)
